# Formation of Zn- and Fe-sulfides near hydrothermal vents at the Eastern Lau Spreading Center: implications for sulfide bioavailability to chemoautotrophs

**DOI:** 10.1186/1467-4866-9-6

**Published:** 2008-05-19

**Authors:** Heileen Hsu-Kim, Katherine M Mullaugh, Jeffrey J Tsang, Mustafa Yucel, George W Luther

**Affiliations:** 1 Civil & Environmental Engineering, Duke University, Box 90287, Durham, NC 27708, USA; 2 College of Marine and Earth Studies, University of Delaware, 700 Pilottown Rd., Lewes, DE 19958, USA

## Abstract

**Background:**

The speciation of dissolved sulfide in the water immediately surrounding deep-ocean hydrothermal vents is critical to chemoautotrophic organisms that are the primary producers of these ecosystems. The objective of this research was to identify the role of Zn and Fe for controlling the speciation of sulfide in the hydrothermal vent fields at the Eastern Lau Spreading Center (ELSC) in the southern Pacific Ocean. Compared to other well-studied hydrothermal systems in the Pacific, the ELSC is notable for unique ridge characteristics and gradients over short distances along the north-south ridge axis.

**Results:**

In June 2005, diffuse-flow (< 50°C) and high-temperature (> 250°C) vent fluids were collected from four field sites along the ELSC ridge axis. Total and filtered Zn and Fe concentrations were quantified in the vent fluid samples using voltammetric and spectrometric analyses. The results indicated north-to-south variability in vent fluid composition. In the high temperature vent fluids, the ratio of total Fe to total Zn varied from 39 at Kilo Moana, the most northern site, to less than 7 at the other three sites. The concentrations of total Zn, Fe, and acid-volatile sulfide indicated that oversaturation and precipitation of sphalerite (ZnS_(s)_) and pyrite (FeS_2(s)_) were possible during cooling of the vent fluids as they mixed with the surrounding seawater. In contrast, most samples were undersaturated with respect to mackinawite (FeS_(s)_). The reactivity of Zn(II) in the filtered samples was tested by adding Cu(II) to the samples to induce metal-exchange reactions. In a portion of the samples, the concentration of labile Zn^2+ ^increased after the addition of Cu(II), indicating the presence of strongly-bound Zn(II) species such as ZnS clusters and nanoparticles.

**Conclusion:**

Results of this study suggest that Zn is important to sulfide speciation at ELSC vent habitats, particularly at the southern sites where Zn concentrations increase relative to Fe. As the hydrothermal fluids mix with the ambient seawater, Zn-sulfide clusters and nanoparticles are likely preventing sulfide oxidation by O_2 _and reducing bioavailability of S(-II) to organisms.

## Background

Deep-sea hydrothermal vents in the Pacific Ocean support productive ecosystems that rely on symbiotic relationships between chemoautotrophic microorganisms and macroinvertebrates [1,2]. Many of these chemoautotrophs utilize inorganic sulfide as the electron source for carbon fixation. As a result, primary productivity in these ecosystems can be controlled by sulfide that enters the surrounding seawater environment from hydrothermal fluid release [3,4]. The concentration, speciation, and subsequent bioavailability of sulfide in the hydrothermal fluids change rapidly as warm, highly reducing vent fluids mix with cold, oxygenated seawater. These chemical gradients can ultimately govern the structure of the macroinvertebrate communities. For example, previous research at the East Pacific Rise (EPR) (9°50'N) has demonstrated that macrofaunal organisms (e.g, tubeworms) were located near direct sources of free sulfide (H_2_S/HS^-^) because they hosted sulfide-reliant symbiotic microorganisms [3]. Macrofauna that did not rely on these chemosynthetic symbionts thrived in habitats where excess Fe(II) reduced the bioavailability (and toxicity) of S(-II) through the formation of FeS clusters and particles [3].

**Figure 1:**
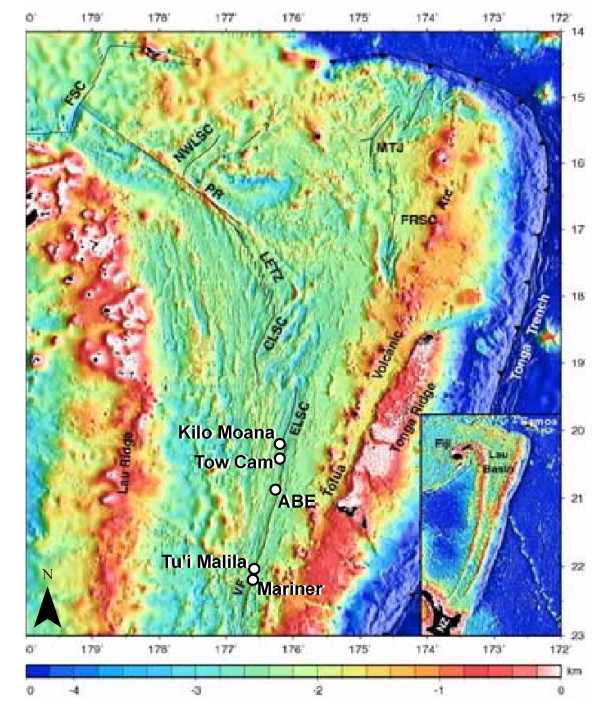
Locations of the four hydrothermal sampling sites during the June 2005 expedition to the Eastern Lau Spreading Center (ELSC) in the Lau Basin, southwest Pacific Ocean: Kilo Moana, Tow Cam, ABE, and Tu'i Malila. Modified from Zellmer and Taylor [46].

The Lau Basin in the southwest Pacific is the site of a back-arc spreading center in which hydrothermal geochemistry and ecology are distinct from EPR and other well-studied vent sites in the Pacific Ocean [5]. The Eastern Lau Spreading Center (ELSC), located within the basin (from 19°20'S to 22°45'S) (Figure 1), exhibits a wide-ranging variation in seafloor spreading rates, ridge morphology, ecological communities, and vent fluid chemistry [6-9]. In particular, large gradients occur over relatively short distances along the north-to-south axis of the ELSC. Northern vent sites along the ELSC (such as Kilo Moana and Tow Cam in Figure 1) are hosted by basalt lavas whereas the southern sites (ABE, Tu'i Malila, and Mariner) are hosted by andesite lavas [10-12]. Sea floor spreading rates decrease by a factor of two from north to south [6]. Recent studies also indicate variability in macrobiological diversity in the diffuse flow regimes along the ELSC ridge axis [13].

The relationships between water chemistry and biological community structure have not been closely studied at the ELSC as they have for EPR. However, results from expeditions to the Lau Basin in the 1980's indicated that the chemical composition and biological community diversity near the ELSC vents were uniquely different from well-studied sites at EPR. For example, macrobiological communities containing sulfide-reliant endosymbionts at the Lau Basin vent sites were distinctive for the diversity of snail species, amongst the most diverse known for deep-sea hydrothermal communities [9]. Tubeworm species such as *Riftia pachytila*, which host sulfide-reliant endosymbionts and are ubiquitous in eastern Pacific vent sites, were apparently non-existent in the Lau vent sites [14].

These biological differences may be partly caused by the unique chemical composition of vent fluids in the Lau Basin. In previous observations at the ELSC, the vent fluids contained sulfide-complexing metals such as Fe, Mn, Cu, Zn, Cd and Pb that were orders of magnitude higher in concentration compared to vent fluids at EPR [7,8]. In particular, the concentration of Zn ranged between 1200 and 3100 μmol kg^-1^, which was approximately equal to the concentration of Fe [8]. The presence of Zn and other metals is important to the availability of dissolved sulfide because precipitation reactions occur if the vent fluids are oversaturated with respect to metal-sulfide minerals. Precipitation in hydrothermal fluids is demonstrated by the deposition of pyrite, sphalerite, and other sulfide-containing minerals in black smoker chimneys and near diffuse flow sources [15]. During precipitation reactions, metal sulfide clusters and nanoparticles are chemical precursors to crystalline forms of the mineral [16]. ZnS-containing minerals and ZnS complexes are thermodynamically more stable than the respective FeS species by several orders of magnitude [17,18]. Therefore, dissolved sulfide speciation in the seawater near ELSC vents may be thermodynamically controlled by the formation of ZnS clusters and particles, rather than FeS. The dominant species of sulfide as the hydrothermal fluids mix with the surrounding seawater may be critical for the macrofaunal habitats that exist at diffuse flow sources and along the sides of black smoker chimneys in the ELSC [5].

The objective of this research was to identify the role of both Zn and Fe for sulfide speciation in vent and diffuse flow fluids in the ELSC. In the 2005 expedition to the Lau Basin, water samples containing high temperature (> 250°C) and diffuse flow (< 50°C) vent fluids were collected from active vents located in the northern, basalt regions and the southern, andesite regions of the ELSC. These samples were analyzed for Zn, Fe and sulfide content. The results of this study indicated that both Fe and Zn are important to sulfide speciation near the Lau hydrothermal sources, particularly in the southern section of the ELSC. This study is the first to investigate the role of zinc for controlling aqueous sulfide speciation at deep-sea hydrothermal vents.

## Methods

### Materials

All chemicals and materials utilized for this research were purchased from Fisher Scientific and were ACS reagent grade, unless otherwise noted. Deionized water was used to prepare all stock solutions. Trace-metal grade acids were used for pH adjustments and acid-cleaning of containers. A NaOH stock solution (1 N) was cleaned of trace metal impurities by adding MgCl_2 _(approximately 5 g added to 500 mL) and filtering the Mg-hydroxide precipitates from the NaOH stock using 0.1 μm syringe filters (Whatman). Ultra high purity argon (Ar) or nitrogen (N_2_) gases were used as needed for purging steps.

Individual metal calibration stocks solutions were prepared by dissolving either 10 mM Zn(NO_3_)_2_•H_2_O or 5.0 mM Cu(NO_3_)_2 _in 0.1 M HNO_3_. Fe(II) standards were prepared by dissolving 10 mM FeCl_2 _in 0.1 M HCl that was purged with N_2_. Reagents for Fe(II) analyses included individual solutions of 5.0 mM ferrozine dissolved in 2.5 M ammonium acetate and 0.2 M NH_2_OH•HCl dissolved in 0.1 M HCl. Sulfide stock solutions were prepared by dissolving crystals of Na_2_S•9H_2_O (rinsed with deionized water and dried prior to weighing) in N_2_-purged deionized water. Sulfide stock solutions were utilized within 1 day. Reagents for the preservation of sulfide-containing samples included 0.2 M zinc acetate and 0.5 M NaOH.

All sample containers were acid-cleaned by soaking overnight in 1 N HCl (trace metal grade) followed by three-times rinse with deionized water. The sample bottles were cleaned in a positive-pressure clean room or in a HEPA-filtered laminar flow hood and were stored in double plastic zip bags. All samples were stored in 2-mL polycarbonate vials or 50-mL polypropylene centrifuge tubes. Samples that were preserved on-board were stored at 4°C until analysis on-shore. Polycarbonate membrane filters (0.2 μm Millipore Isopore GTTP) fitted with acid-cleaned Swinnex filter holders were used for filtration of vent fluid samples.

### Field study site

The field expedition to the Lau Basin in June 2005 focused on aquatic and biological sampling at four site stations in the ELSC (Figure 1). The stations Kilo Moana (KM) and Tow Cam (TC) are located along the northern ELSC where basalt mineralogy dominates the region. ABE and Tu'i Malila (TM) are located in the southern, andesite-hosted region of the ELSC.

### Sample collection

Dives at each station occurred during June 2005 with the ROV Jason II. Concentrated, high-temperature hydrothermal fluids and diffuse, low-temperature vent fluids were collected at all sites using titanium (Majors) samplers (summarized in Table 1). These were collected by the ROV *Jason II *pilots, who waited for a constant temperature reading before triggering the samplers. The Majors were retrieved on-board within 12 hr after collection. Samples were immediately filtered and preserved as needed. Supplementary information listing date, longitude and latitude of sample sites are located in Additional file 1.

**Table 1:** Concentrations of acid-volatile sulfide (AVS), Fe and Zn in the unfiltered and 0.2-μm filtered samples collected at hydrothermal vents of the Eastern Lau Spreading Center (June 2005).

					AVS, μM		Fe, μM		Zn, μM	
					
Station	Sample ID	Sample Site Description	Temp. °C	pH	unfiltered	filtered	unfiltered	filtered	unfiltered	filtered
Kilo Moana	KM-154-2	Diffuse flow near mussel bed	12.3	7.0	52.3	16	16	15	< 0.3	< 0.3
	KM-155-2	Black smoker	178	3.7	2710	1430	1430	1400	90	< 0.3
	KM-155-3	Black smoker	252	5.8	148	202	202	280	13	< 0.3
	KM-156-1	Black smoker	258	4.6	2230	1570	1570	1560	22	< 0.3
	KM-156-3	Black smoker	314	3.6	2250	1580	1580	1480	85	< 0.3
	KM-164-2	Black smoker	301	5.2	1190	852	852	780	12	< 0.3
Tow Cam	TC-157-1	Black smoker	325	4.1	3990	234	234	230	33	< 0.3
	TC-157-2	Black smoker	310	4.3	N/A	230	230	230	43	< 0.3
	TC-159-1	Diffuse Flow	35	6.6	89.3	5.6	5.6	2.5	< 0.3	< 0.3
	TC-159-2	Diffuse Flow	35	6.5	172	5.9	5.9	5.6	< 0.3	< 0.3
ABE	ABE-160-1	Clear fluid from a broken spire	153	5.6	359	27.6	27.6	33	< 0.3	< 0.3
	ABE-160-2	Black smoker	295	4.2	1370	235	235	240	39	< 0.3
	ABE-160-3	Black smoker	310	4.2	1540	158	158	180	42	< 0.3
	ABE-160-4	Black smoker	305	4.4	1570	149	149	150	36	< 0.3
Tu'i Malila	TM-161-1	Focused diffuse flow, clear	45	6.1	161	1.1	1.1	0.80	< 0.3	< 0.3
	TM-161-2	Clear vent fluid	295	4.2	760	124	124	130	42	1.5
	TM-161-3	Focused diffuse flow, clear	45	6.2	148	2.3	2.3	1.9	< 0.3	< 0.3
	TM-161-4	Clear vent fluid	267	4.5	804	165	165	163	20	0.5

### Sample analyses

Fe and pH analyses were conducted in the aqueous samples as soon as they were brought on-board the ship. Ferrous iron (Fe^2+^) was quantified by addition of the ferrozine-ammonium acetate mixture to filtered samples (final concentration of 2.5 mM ferrozine) [19]. Total iron concentration (Fe(II) and Fe(III)) was quantified by first reducing the filtered and unfiltered samples in 17 mM NH_2_OH•HCl and then allowing the sample to react overnight prior to addition of the ferrozine-acetate reagent. The Fe(II)-ferrozine derivative was detected by UV absorbance at 560 nm (DLK-1000 filter spectrophotometer).

For analysis of acid-volatile sulfide (AVS), filtered and unfiltered samples were preserved on-board by zinc acetate and NaOH (final concentrations of 20 mM and 5 mM, respectively). AVS was analyzed after the cruise by thawing and acidifying the sample to 1.5 M HCl in sealed glass vessels. The resulting H_2_S was purged from the vessel with Ar gas for at least 60 minutes and trapped as HS^- ^in 20.0 mL trace-metal clean 0.1 N NaOH. Extraction of AVS includes H_2_S, HS^-^, and amorphous metal-sulfide species such as FeS and ZnS [20]. Recovery tests were conducted with sulfide standards prior to sample analyses and confirmed > 95% yield of sulfide by this procedure. The concentration of AVS was measured in the NaOH trapping solution by cathodic stripping voltammetry on a hanging mercury drop electrode (Princeton Applied Research Model 303A). A saturated calomel electrode (SCE) was used as the reference electrode. When necessary, sulfide deposition steps were conducted for 60 s at -0.30 V prior to scanning the electrode potential from -0.30 V to -1.80 V in cyclic voltammetry mode (1.00 V/s scan rate). For samples with high levels of sulfide, the deposition step was not needed. Sulfide was detected by the resultant current peak height at -0.60 V. The limit of detection for AVS was less than 10 nM (corresponding to 60 s deposition).

Aliquots of vent fluid samples from Tu'i Malila were preserved and analyzed for dissolved organic carbon (DOC) concentration. These samples were filtered utilizing glass fiber filters (Whatman GF/F) and a glass vacuum filtration apparatus. All filtration devices and sample containers for DOC analysis were baked overnight at 400°C prior to the cruise. Samples for DOC analysis were preserved by acidification to 0.1 M HCl, subsequently sealed in glass ampules, and stored frozen until analysis. DOC analysis occurred on-shore by combustion catalytic oxidation-infrared spectroscopy (Shimadzu TOC analyzer). Ultrapure water (> 18 MΩ-cm, Millipore MilliQ) was used to rinse all DOC sample containers and dilute DOC standard solutions.

For total Zn analyses, separate aliquots of the unfiltered and filtered (< 0.2 μm) vent fluid samples were preserved on-board by acidification to 0.1 M HNO_3_. The concentration of Zn was measured in the unfiltered samples after the cruise by diluting the samples by a factor of 5 in 0.1 M HNO_3_, and quantification by inductively-coupled plasma optical emission spectrometry (Horiba-Jobin-Yvon). After accounting for dilution, our limit of detection for total Zn in the vent samples was 0.3 μM.

The speciation of dissolved Zn(II) was assessed by saving separate aliquots of 0.2-μm filtered samples that had no additional amendments (*e.g*., no acid added). These samples were stored in 50-mL polypropylene centrifuge tubes at 4°C. After the cruise, the concentration of Zn^2+ ^and other labile species were quantified by anodic stripping voltammetry (ASV). Depositions were conducted at -1.3 V for either 10 s or 120 s. The concentration of Zn was quantified by stripping in square wave mode from -1.3 V to -0.1 V (0.025 V pulse height, 0.20 V/s scan rate) and measuring the resultant current peak height at -1.0 V. Zn peak height was converted to concentration according to an external calibration of the Zn stock added to seawater. In order to prevent metal contamination carry-over between samples, the HMDE sample holder, Ar-purging tube and stirrer were rinsed with 0.1 M HCl followed by three-times rinse with deionized water. The limit of detection was less than 5 nM, corresponding to the 120 s deposition time. Zn(II) concentration that was quantified according to this method is referred in this study as ASV-labile Zn.

### ZnS model samples

Buffered aqueous samples containing Zn and sulfide were prepared in the laboratory in order to investigate the possible mechanisms that stabilize ZnS clusters and nanoparticles in solution. The model samples were prepared by dissolving 2 μM Zn(NO_3_)_2 _and 2 μM Na_2_S•9H_2_O in air-saturated deionized water buffered to pH 7.6 by 4.0 mM 4-(2-hydroxyethyl)piperazine-1-ethanesulfonic acid (HEPES) in 0.1 M KCl. The role of organic acids during ZnS precipitation was also tested by preparing separate aliquots of the model samples with L-cysteine (CYS). In these solutions, CYS was added to a concentration of 2.5 μM prior to the additions of zinc and sulfide. After allowing 30 min for the formation of amorphous ZnS, the solutions were filtered to < 0.2 μm (Whatman Isopore GTTP). The concentration of ASV-labile Zn was quantified after filtration, as described in the previous section.

### Cu(II)-exchange studies

ZnS clusters and nanoparticles are inert (*i.e*., not detected) during ASV analysis [21]. The potential formation of ZnS clusters and nanoparticles in the vent fluid samples was further assessed by testing their reactivity in the presence of Cu(II). The model ZnS solutions and the vent fluid samples were amended with Cu^2+ ^in order to determine whether metal-exchange reactions would occur. Addition of Cu(II) would destabilize ZnS and release Zn^2+ ^into solution by displacing Zn to form CuS species with reduction of Cu(II) and oxidation of sulfide [22,23]. In aliquots of the filtered vent fluid samples and in the model ZnS solutions, Cu^2+ ^was added to final Cu(II) concentrations between 0.2 and 0.5 μM. Each air-saturated sample was allowed to react for at least 10 minutes, subsequently purged with Ar, and analyzed for any increase in ASV-labile Zn.

## Results and Discussion

### Diffuse vs. high temperature vent fluid chemistry

**Figure 2:**
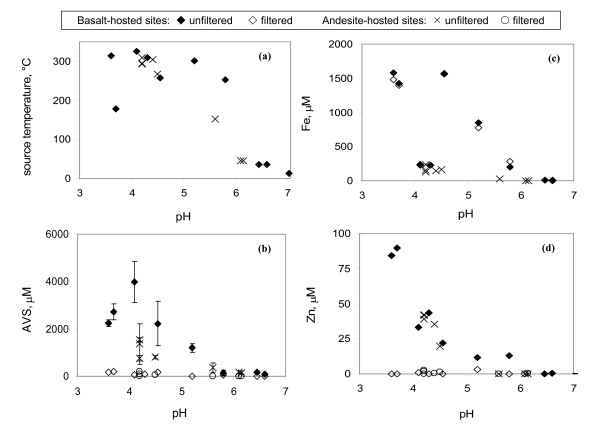
a) Temperature, b) acid-volatile sulfide (AVS), c) total Fe(II)+Fe(III), and d) total Zn as a function of pH in unfiltered and filtered vent fluids collected from ELSC vent fields located in basalt-hosted and andesite-hosted sites.

Quantification of Fe, Zn and sulfide concentrations in diffuse-flow (< 50°C) and high-temperature (> 250°C) vent fluids provided insight to the speciation of sulfide near these hydrothermal sources. At all field sites, the concentration of Fe, Zn and sulfide was dependent upon the temperature and pH of the original source. High temperature vent fluids were between pH 3.7 and 5.8; in contrast, the pH of the diffuse-flow samples ranged between pH 6.1 and 7.0 (Figure 2a). Samples collected from high temperature/low pH sources also contained the highest concentrations of acid-volatile sulfide, Fe and Zn (Figures 2b, c and 2d). These differences demonstrated that our fluid samples included a range of composition from pure hydrothermal vent fluid to ambient seawater. In some high temperature fluid samples, some mixing of pure hydrothermal fluid and ambient seawater occurred due to entrainment of seawater during the sampling process. Thus, the measured S(-II), Fe and Zn concentrations do not represent end-member concentrations (e.g., pure hydrothermal fluid). Instead the samples represented mixtures of vent fluid and seawater that are relevant for assessing exposure to macroinvertebrate organisms. In the ELSC, these communities were observed to exist directly over diffuse venting sources or attached to the outside of black smoker chimneys (where high temperature fluids can permeate through the chimney wall) [5].

### North-to-south variability

**Figure 3:**
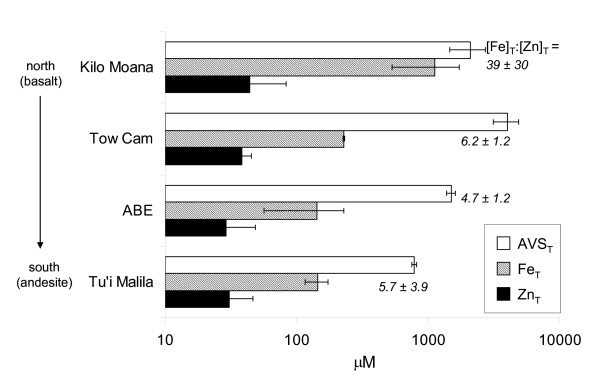
Total acid-volatile sulfide (AVS), total Fe and total Zn concentrations in high temperature (> 250°C) vent fluid samples collected along the north-to-south ELSC ridge axis. The bars represent average ± one standard deviation (n = 2 to 5). The average [Fe]_T_:[Zn]_T _ratio is also noted for each site.

The ELSC is notable for north-to-south gradients in seafloor geology, ridge morphology and hydrothermal activity [6-9]. Results of this study indicated that Fe, Zn and sulfide concentrations in the high temperature vent fluids (> 250°C) decreased from north to south at the four sites (Table 1 and Figure 3). In particular, the average total Fe concentration from high temperature vents at Kilo Moana was 1126 ± 597 μM (n = 5), almost ten times greater than vent fluids from the other sites (ranging from 124 to 234 μM total Fe) (Table 1). Total Zn and sulfide concentrations also decreased from north to south (Figure 3). These data coincide with the shift in lava composition along the ridge axis (from basalt-hosted to andesite-hosted venting sites). We note, however, that these observations are based on the averages of relatively small sample sizes (n = 2–5 at each site) and additional samples are needed to confirm this north-to-south trend.

The data also show spatial trends for the relative concentrations of Fe, Zn and S(-II). In particular, the average ratio of total Fe to total Zn in the high-temperature fluids was 39 (± 30, n = 5) at the most northern site, Kilo Moana (Figure 3). The observed Fe:Zn ratios at Kilo Moana were similar to the composition of high temperature vent fluids at EPR and Juan de Fuca Ridge in the eastern Pacific, where Fe:Zn ratios are typically 10 or greater [24-26]. In contrast, the Fe:Zn ratio was lower at the three other sites at ELSC (ranging from 4.7 to 6.2). Other researchers have also demonstrated relatively low Fe:Zn ratios in vent fluids at the southern ELSC compared to hydrothermal sites in the eastern Pacific [8]. These observations indicate the importance of Zn in the southern ELSC for the sequestration of sulfide near hydrothermal sources. The half-life for oxidation of free sulfide in the presence of dissolved oxygen is approximately one day [27,28]. However, when sulfide is coordinated to Zn, the half-life for sulfide oxidation is approximately 22 days [22]. The reduced presence of Fe relative to Zn at Tow Cam, ABE, and Tu'i Malila highlights the need to consider Zn-sulfides for sulfide speciation (and its subsequent bioavailability) in the ambient seawater surrounding the hydrothermal fluid release points.

### FeS_(s) _and ZnS_(s) _saturation index

**Figure 4:**
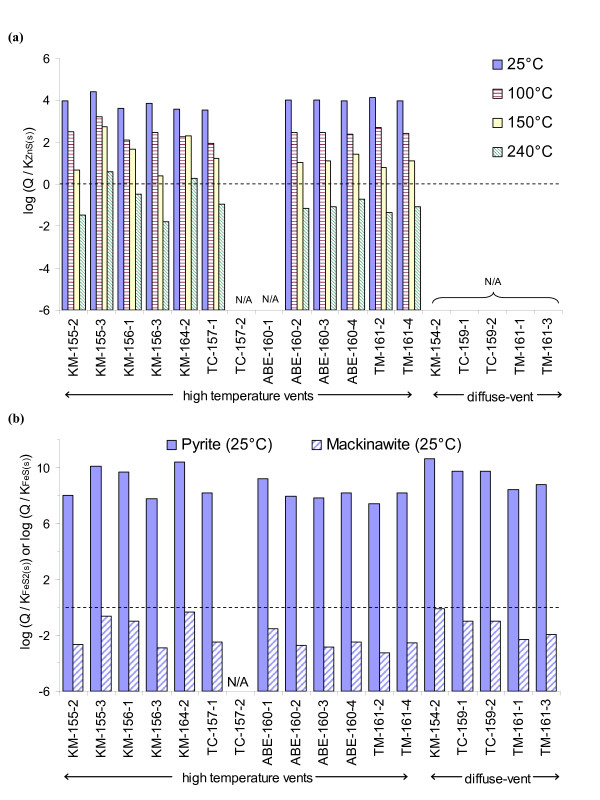
Calculated saturation index for each sample using the pH, Fe, Zn, and acid-volatile sulfide (AVS) concentrations measured in unfiltered samples: **a) **ZnS_(s) _(sphalerite) saturation index at four temperatures (1 bar); **b) **FeS_2(s) _(pyrite) and FeS_(s) _(mackinawite) saturation indices (25°C, 1 bar). Calculations at 25°C, 1 bar, were conducted using the thermodynamic database in MINEQL+. Saturation indices corresponding to 100°C, 150°C, and 240°C were calculated using equilibrium constants listed in Table 2. The calculations assumed 0.55 M Cl^- ^and ionic strength corrections by the Davies equation for I = 0.5 M. N/A denotes that the total AVS was not available or total Zn concentration was below the analytical detection limit (< 0.3 μM) by ICP-OES.

To assess the potential for Fe- and Zn-sulfide precipitation in the vent fluid samples, the saturation indices for mackinawite FeS_(s)_, pyrite FeS_2(s)_, and sphalerite ZnS_(s) _were estimated from the measured concentrations of Fe, Zn and acid-volatile sulfide in the vent fluid samples. The objective of these calculations was to assess the thermodynamic potential for precipitation as the pure vent fluids were released to the surrounding seawater. Thus, the saturation indices were based on concentrations in the unfiltered samples, assuming that the metals and sulfides were dissolved in the original vent fluids at the point of release. The calculations were conducted using MINEQL+ [29] for 25°C, 1 bar. The input parameters for these calculations were the measured pH, Fe, Zn, and sulfide (i.e., acid volatile sulfide) concentrations of the unfiltered samples (Table 1). The vent fluid samples were assumed to contain dissolved Cl^- ^as in seawater (0.55 M Cl^-^), a concentration similar to those measured by other researchers in Lau Basin hydrothermal fluids (ranging from 0.5 M to 0.8 M) [7,8,30]. The Davies equation was used for ionic strength corrections (assuming I = 0.5 M). Sphalerite saturation indices (Figure 4) were also calculated at higher temperatures (100°C, 150°C, 240°C) using the equilibrium constants from Hayashi *et al*. [31] (and references therein) and listed in Table 2.

**Table 2:** Stability constants for equilibrium reactions utilized to calculate saturation indices for ZnS_(s) _(sphalerite), FeS_(s) _(mackinawite) and FeS_2(s) _(pyrite).

	log K (I = 0)			
	
	25°C^(1)^	100°C^(2)^	150°C^(2)^	240°C^(2)^
H_2_O_(l) _⇔ H^+ ^+ OH^-^	-14.0	-12.22	-11.59	-11.08
H_2_S_(aq) _⇔ HS^- ^+ H^+^	-7.0	-6.38	-7.62	-7.57
HS^- ^⇔ S^2- ^+ H^+^	-17.0	-16.01	-15.51	-14.83
				
Zn^2+ ^+ Cl^- ^⇔ ZnCl^+^	0.4	1.22	2.05	4.13
Zn^2+ ^+ 2Cl^- ^⇔ ZnCl_2_^0^	0.6	1.87	3.08	5.29
Zn^2+ ^+ 3Cl^- ^⇔ ZnCl_3_^-^	0.5	2.32	3.74	6.42
Zn^2+ ^+ 4Cl^- ^⇔ ZnCl_4_^2-^	0.19	1.45	2.61	5.8
				
Zn^2+ ^+ 2HS^- ^⇔ Zn(HS)_2_^0^	12.82	11.94	12.05	13.26
Zn^2+ ^+ 3HS^- ^⇔ Zn(HS)_3_^-^	16.1	13.21	13.4	15.43
Zn^2+ ^+ 2HS^- ^⇔ ZnS(HS)^- ^+ H^+^	6.81	6.14	5.89	5.99
Zn^2+ ^+ 3HS^- ^⇔ ZnS(HS)_2_^2- ^+ H^+^	6.12	5.56	5.22	N/A
Zn^2+ ^+ 2HS^- ^+ Cl^- ^⇔ Zn(HS)_2_Cl^-^	N/A	13.16	13.42	14.33
				
Zn^2+ ^+ HS^- ^⇔ ZnS_(s) _+ H^+^	11.45	10.59	10.4	10.79
				
Fe^2+ ^+ 2HS^- ^⇔ Fe(HS)_2_^0^	8.95			
Fe^2+ ^+ 3HS^- ^⇔ Zn(HS)_3_^-^	10.99			
Fe^2+ ^+ HS^- ^⇔ FeS_(s), mackinawite _+ H^+^	3.6			
Fe^2+ ^+ HS^- ^+ S^0^_(s) _⇔ FeS_2(s), pyrite _+ H^+^	14.2^(3)^			

^(1) ^From MINEQL+ thermodynamic database [29].

^(2) ^From Hayashi *et al*. [33] [31].

^(3) ^From Rickard and Luther [35].

In all of the vent samples where Zn was detected, the ZnS_(s)_-sphalerite saturation index at 25°C (1 bar) was greater than zero (Figure 4a), indicating that ZnS_(s) _precipitation was favorable at room temperature conditions. These solubility calculations, however, are strongly dependent upon temperature. Under hydrothermal temperatures (> 200°C), for example, the stability of dissolved Zn-chloride-bisulfide species increase significantly compared to room temperature conditions, leading to increased solubility of sphalerite [32,33,31,30]. Thus, precipitation of metal-sulfides at hydrothermal vents is largely driven by cooling temperatures and increasing pH as the vent fluids mix with the surrounding seawater [32]. Our estimated saturation indices for ZnS_(s) _confirm the temperature dependence of the ZnS_(s) _solubility. According to our calculations, dissolved Zn-chloride and -bisulfide complexes were the primary Zn species at the temperature of the vent fluid source. Oversaturation of ZnS_(s) _may be occurring; however, only as the fluids cool when they enter the surrounding seawater to temperatures of 150°C or less.

For pyrite (FeS_2(s)_), the estimated saturation indices at 25°C (1 bar) in all the samples were greater than zero, sometimes by several orders of magnitude (Figure 4b). These calculations indicate the potential for FeS_2(s) _formation at all of the sites, particularly during cooling of the hydrothermal fluids. In contrast, all of the vent fluid samples were undersaturated or near equilibrium with respect to mackinawite at 25°C (Figure 4b). The solubility of FeS_(s) _and FeS_2(s) _also increased under hydrothermal temperature [32]. This temperature dependence would not change the saturation condition of FeS_(s) _(i.e., undersaturated at temperature > 25°C in all samples). The mechanisms for FeS_2(s) _formation generally involve soluble forms of FeS (material that passes through < 0.2 μm filters) as a reactant with either H_2_S_(aq) _or polysulfides [34,35]. Thus, precipitation of FeS_2(s) _may be kinetically-controlled by formation of soluble FeS under these conditions.

The solubility of metal-sulfides also changes with pressure. However, the solubility constants for sphalerite, pyrite and other metal-sulfides generally increase by less than one log unit when pressure increases from 1 bar to 200 bar (the approximate pressure at the ELSC vent sites) [32]. Thus, the saturation indices of ZnS_(s)_, FeS_(s)_, and FeS_2(s) _are only weakly dependent on pressure relative to temperature.

The saturation index calculations utilized pH measurements taken on-board the ship (after the samples cooled to near room temperature). The decrease in temperature and pressure likely altered the pH of the samples. Ding and Seyfried [36] reported that the in-situ pH of high-temperature vents fluid is approximately 0.5–2.0 pH units greater than measurements collected at 25°C and 1 bar. Thus, our pH measurements are likely to be an underestimate of in-situ conditions. Nevertheless, an increase of pH (under in-situ conditions) would result in increasing saturation indices, suggesting that our values in Figure 4 are underestimations.

### ZnS clusters and nanoparticles

Precipitation of metal-sulfides such as ZnS_(s) _involves clusters and nanoparticles as precursors to the crystalline mineral phase [16]. ZnS clusters and nanoparticulates are small enough to pass through sample filters (< 0.2 μm); however, they remain inert (*i.e*. not detectable) during analysis by anodic stripping voltammetry [21]. As indicated by our previous research [37], weak to moderately strong Zn^2+^-ligand complexes dissociate during the electrochemical deposition step (at -1.3 V). According to the chelate scale reported by Lewis *et al*. [37], Zn-ligand complexes with stability constant, *K*, less than 10^16.2 ^were capable of dissociating during deposition at -1.3 V. In this case, *K *is defined as:

(1) $$K=\frac{\left\{Zn_{y}L_{x}^{2y-nx}\right\}}{{\left\{Zn^{2+}\right\}}^{y}{\left\{L^{n-}\right\}}^{x}}$$

The mononuclear Zn-sulfide complex, ZnHS(H_2_O)_5_^+^, has a *K *value approximately equal to 10^6.1 ^at 25°C in seawater (where *L*^*n*- ^is HS^-^, as written in Eq. 1) [38]. Therefore, ZnHS(H_2_O)_5_^+ ^complexes should dissociate during the electrochemical deposition step given the conditions in our samples. Multinuclear Zn-sulfide clusters or nanoparticles tend to be more stable and, consequently, are inert during the ASV deposition step [21].

**Figure 5:**
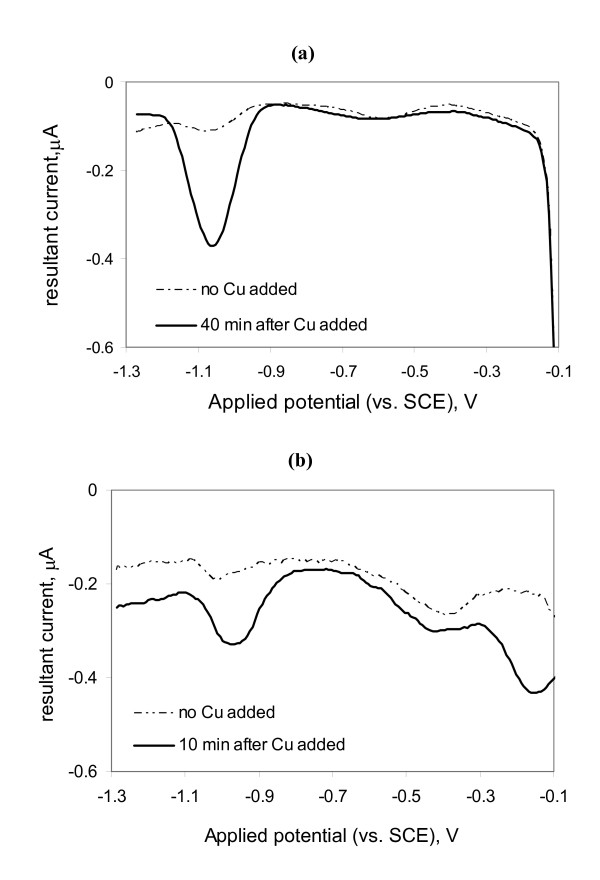
Anodic stripping square wave voltammograms of Ar-purged sample in the absence and presence of 0.5 μM Cu(II). a) 2.0 μM ZnS model solution (0.1 M KCl, pH 7.6). The deposition step occurred for 10 s at -1.3 V. b) Filtered diffuse-flow vent fluid from Tow Cam (TC-159-2), which contained approximately 0.1 μM labile Zn^2+ ^prior to Cu(II) addition. The deposition step occurred for 120 s at -1.3 V. The half-wave potentials (vs. SCE) for the metal analytes are -1.0 V for Zn^2+ ^and -0.18 V for Cu^2+^.

**Figure 6:**
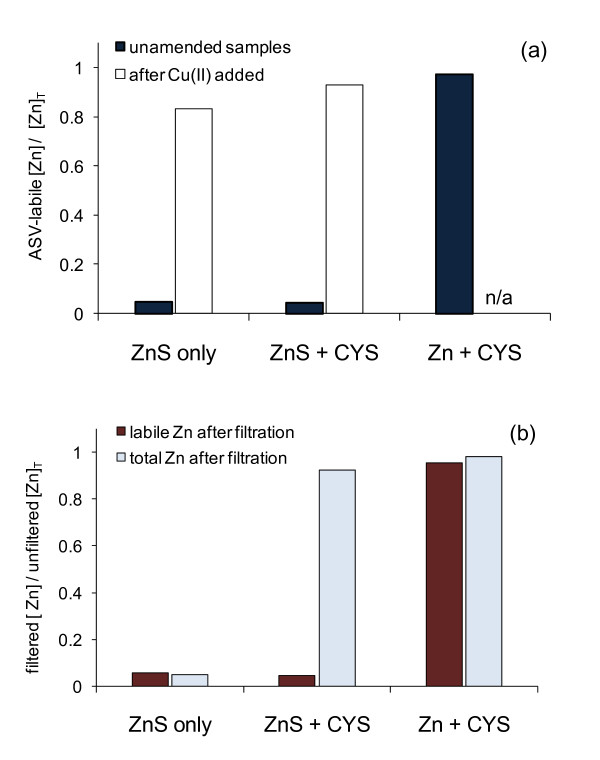
Zn concentration as determined by anodic stripping voltammetry in model Zn-sulfide solutions containing 2 μM ZnS, 2 μM ZnS + 2.5 μM cysteine (CYS), and 2 μM Zn + 2.5 μM CYS (all model solutions prepared in 0.1 M KCl, 4 mM HEPES buffer, pH 7.6). (a) ASV-labile Zn in unfiltered solutions before and after addition of 0.5 μM Cu(II). Addition of Cu(II) caused oxidative dissolution of ZnS; (b) ASV-labile and total Zn quantified in filtered (< 0.2 μm) solutions.

Experiments with the model ZnS solutions demonstrated that the addition of Cu(II) caused the release of Zn^2+ ^into solution, thus providing a possible method for detecting strongly bound Zn species such as ZnS clusters and nanoparticles. As shown in Figure 5a and Figure 6a, the addition of 0.5 μM Cu(II) to the air-saturated model samples of 2.0 μM ZnS (prior to the purging step during ASV analysis) resulted in > 83% recovery of total Zn in solution after 40 min of reaction time. In these model solutions, more Zn(II) was released to solution than expected based on the amount of Cu(II) added, indicating that Cu(II) was involved in catalyzing oxidative dissolution of the Zn-sulfides. Previous researchers have shown that redox active metals such as Cu^2+ ^and Fe(III) can accelerate oxidation of sulfide in air-saturated conditions in which Cu(II)/Cu(I) and Fe(III)/Fe(II) are the electron shuttles between S(-II) and dissolved oxygen [22,39,40]. Furthermore when dissolved sulfide concentrations are equal to or ten times greater than Cu^2+ ^concentrations, the half-life for oxidation ranges from 10 min to 30 min in seawater [22], consistent with our model studies.

**Figure 7:**
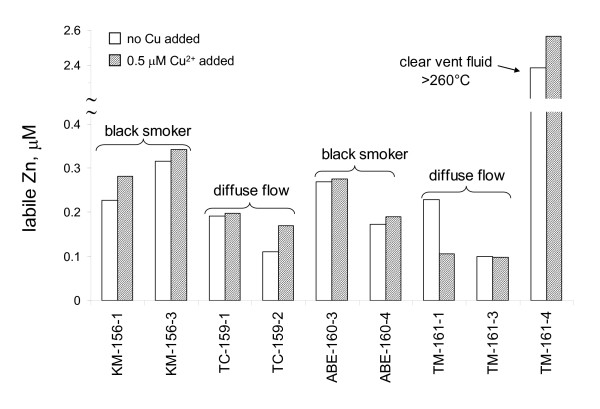
ASV-labile Zn^2+ ^in filtered vent fluid samples collected from the ELSC and in filtered samples amended with 0.5 μM Cu(II) prior to analysis for ASV-labile Zn^2+^.

Similar Cu(II)-addition experiments were conducted with the vent fluid samples (*e.g*., sample #TC-159-2 in Figure 5b). Cu(II) was added to filtered vent fluid samples that contained low but detectable levels of ASV-labile Zn and were allowed to react for 10 to 15 minutes in air-saturated conditions. When the samples were subsequently purged and analyzed by anodic stripping voltammetry, the concentration of labile-Zn slightly increased in most of the samples tested including the high temperature vent fluid samples (Figure 7). Figure 5b shows a treatment where the concentration of added Cu(II) was more than the total Zn(II). Thus, a Cu^2+ ^peak was observed at an electrode potential of -0.18 V. Longer Cu(II) reaction times were probably necessary for complete recovery of Cu-reactive Zn(II), particularly in samples where the added Cu(II) was less than total Zn concentration (*e.g*., sample #TM-161-4). Furthermore, the size and structure of ZnS clusters and nanoparticles were probably changing during the time between sample collection and the Cu(II) exchange measurements in the laboratory (which occurred 2–3 weeks after the cruise). Thus, 'aging' time was important for quantifying the concentration of Zn that was reactive to Cu(II). The results, nevertheless, indicated that strongly-bound species of Zn(II) were present in a subset of the samples and that the Zn(II) was labile only after Cu(II) was added. In all of these samples, the acid-volatile sulfide concentration was greater than the total Zn concentration. Therefore, the Zn^2+ ^released during the Cu-addition experiments was likely bound as ZnS clusters and nanoparticles.

Dissolved organics are known to stabilize nanoparticles and colloids by adsorbing to particle surfaces and preventing aggregation [41-43]. Thiol-containing compounds are also particularly effective capping agents for metal-sulfide clusters and nanoparticles at circumneutral pH by coordinating to the surface of the metal-sulfides and preventing further growth or aggregation of particles [41]. Separate model solutions containing 2.0 μM ZnS were prepared in the presence (and absence) of 2.5 μM cysteine (CYS), a thiol-containing amino acid. According to the Zn^2+ ^and H_2_S concentrations predicted by MINEQL+ [29] for the ZnS-CYS model solutions, the saturation index for ZnS_(s) _(i.e., log *Q/K*_*s*, *ZnS*_) was equal to +5.5, demonstrating oversaturated conditions in the model experiments. When the model ZnS-CYS solutions were filtered, > 92% Zn was recovered in the solution after filtration (Figure 6b), indicating that CYS prevented the formation of ZnS particles larger than the pore size of the filters (0.2 μm). In the absence of CYS, less than 5% of ZnS was recovered after filtration, indicating removal of ZnS particles. Voltammetric analysis of Zn(II) speciation in the filtered ZnS-CYS solution showed that the Zn(II) was inert. This result indicated that Zn was coordinated to S(-II) as ZnS clusters and nanoparticles and was not in the form of Zn-CYS complexes, which are labile during the anodic stripping voltammetry analysis (Figure 6a).

Hydrothermal vent fluids contain dissolved organics that can help to stabilize metal-sulfide clusters. In the high- and low-temperature vent fluids at Tu'i Malila, DOC concentrations were between 60 and 120 μM. DOC concentration in the ambient seawater at the ELSC was not determined; therefore, the possibility of contamination from the sample collection procedure could not be assessed. These results are, nevertheless, consistent with measurements at other sites in the eastern Pacific, where DOC levels in diffuse-flow vents were in excess of those found in the ambient seawater [44,45]. A main source of DOC in diffuse hydrothermal fluids is microbial productivity, which can release amino acids and other biomolecules to the water [44]. These organics may be capping metal-sulfide clusters and decelerating aggregation as precipitation reactions occur. In the samples that contained non-labile Zn species, organics may play an important role for ZnS cluster and nanoparticle stabilization. The inherit oversaturation of ZnS_(s) _coupled with the potential for decreased aggregation from capping organics could have important implications for Zn and sulfide transport and sulfide bioavailability to chemoautotrophs at these hydrothermal systems.

## Conclusion

Results of this study demonstrate the presence of sulfide-complexing metals such as Fe and Zn in hydrothermal fluids of the ELSC. The results also provide insight to the speciation of sulfide that might be available to the surrounding organisms. ZnS_(s) _precipitation was thermodynamically favored at all sites in the ELSC; however, only as hydrothermal fluids cooled after collection. At the low pH (< 4) and high temperatures of vent fluids, dissolved Zn-bisulfide and Zn-chloride species appear to be the major fraction of total Zn(II) based on MINEQL+ calculations. As vent fluids mix with ambient seawater, pH increases as temperature decreases, inducing the formation of ZnS clusters and nanoparticles. Zn speciation analyses and the Cu(II)- reactivity experiments suggested that ZnS clusters and nanoparticles were also present in a subset of the high temperature vent fluid samples after cooling.

The presence of ZnS could provide another pathway, in addition to FeS [3], that would decrease free sulfide (H_2_S and HS^-^) concentration in the surrounding seawater at the ELSC. A decrease in dissolved free sulfide by ZnS complexation and/or precipitation would decrease bioavailability to sulfide-reliant chemoautotrophs and also inhibit the growth of macroinvertebrates that harbor chemoautotrophic endosymbionts (as we observed for Fe-sulfide speciation at vents located at the East Pacific Rise [3]). At the same time, a decrease in free sulfide concentrations could permit other non-chemoautotrophic species to grow (also observed at EPR [3]). Further studies at the southern ELSC should focus on the formation and persistence of FeS, ZnS, and possibly other metal-sulfide clusters and nanoparticles (such as CdS and PbS) that are perhaps stabilized by organic compounds near the vent sources. This information would improve our understanding of the linkages between vent fluid chemistry and vent ecology at the ELSC.

## Authors' contributions

HH carried out sample collection, performed chemical analyses, interpreted data, and drafted the manuscript. KMM performed the AVS measurements and assisted with data interpretation and the manuscript. JTT assisted with sample collection and performed the Fe measurements. MY assisted with solubility calculations and manuscript preparation. GWL assisted with the experimental design, led the coordination of the study, and participated in drafting the manuscript.

## Supplementary Material

Additional file 1 Location and collection data of vent fluid samples. The table includes longitude, latitude, and date for each vent fluid collected. Click here for file
