# Supplementary material for: Formation of Zn- and Fe-sulfides near hydrothermal vents at the Eastern Lau Spreading Center: implications for sulfide bioavailability to chemoautotrophs
Source: Geochem Trans. 2008 May 19;9:6. doi: 10.1186/1467-4866-9-6 (PMC2396607; doi:10.1186/1467-4866-9-6)
Supplement: Additional file 1 — Location and collection data of vent fluid samples. The table includes longitude, latitude, and date for each vent fluid collected. [file 1467-4866-9-6-S1.pdf]

Supplementary Table 1. Locations and collection date of each vent fluid sample

Station	Sampling date	Sample ID	Latitude	Longitude
Kilo Moana	Jun-12-05	KM-154-2	20 3.232946 S	176 8.011579 W
	Jun-14-05	KM-155-2	20 3.229245 S	176 8.007376 W
		KM-155-3	20 3.115110 S	176 8.096364 W
	Jun-15-05	KM-156-1	20 3.235369 S	176 8.006933 W
		KM-156-3	20 3.236395 S	176 8.006927 W
	Jun-26-05	KM-164-2	20 3.153036 S	176 8.028189 W
Tow Cam	Jun-15-05	TC-157-1	20 5.987972 S	176 8.182862 W
		TC-157-2	20 5.987292 S	176 8.183047 W
	Jun-17-05	TC-159-1	20 5.998519 S	176 8.218040 W
		TC-159-2	20 5.998173 S	176 8.217600 W
ABE	Jun-18-05	ABE-160-1	20 45.653674 S	176 11.461254 W
		ABE-160-2	20 45.653513 S	176 11.445403 W
		ABE-160-3	20 45.656731 S	176 11.442188 W
		ABE-160-4	20 45.931271 S	176 11.580822 W
Tu’i Malila	Jun-21-05	TM-161-1	21 59.358871 S	176 34.048289 W
		TM-161-2	21 59.405976 S	176 34.135512 W
		TM-161-3	21 59.355964 S	176 34.067263 W
		TM-161-4	21 59.277934 S	176 34.063783 W
